# India’s Battle against COVID-19: Progress and Challenges

**DOI:** 10.4269/ajtmh.20-0992

**Published:** 2020-08-24

**Authors:** Ramanan Laxminarayan, Shahid Jameel, Swarup Sarkar

**Affiliations:** 1 Center for Disease Dynamics, Economics & Policy, New Delhi, India;; 2 Princeton University, Princeton, New Jersey;; 3 The Wellcome Trust/DBT India Alliance, New Delhi, India;; 4 Independent Consultant, New Delhi, India

## INTRODUCTION

India was fortunate not to be among the first countries hit by COVID-19. The first reported case of infection with the SARS-CoV-2, the virus that causes COVID-19, in India was reported on January 30, 2020 in an Indian student evacuated from Wuhan, and the first death was reported on March 12, 2020. Although it is possible that the SARS-CoV-2 was circulating in India earlier, the first known secondary transmission of the virus within India was reported only in early March.

Even when the case load was low, India was predicted to be at high risk for COVID-19 for a number of reasons. A dense population, especially in urban settings, could exacerbate the spread of SARS-CoV-2. The Indian population has high rates of uncontrolled hypertension^1^ and diabetes,^2^ both of which have been shown to be risk factors for severe COVID-19 and mortality. Although children were found less likely to be infected in China^3^ and other countries, it was unclear how children in India, who have high levels of stunting and early exposure to infections, would fare against SARS-CoV-2.^4^ On the plus side, it was hypothesized that India’s relatively young population—65% of India’s population is younger than 35 years, and only 6% is older than 65 years—would be spared high mortality from a disease that targets the elderly. In comparison, 22% of the population of Italy and 10% of that of China are older than 65 years.

## COVID-19 RESPONSE AND THE STATUS OF THE PANDEMIC IN INDIA

How did India respond and how has it fared? The Indian government responded to COVID-19 with temperature screening of incoming passengers on flights from East Asia. Large public events, including one to greet U.S. President Donald Trump, were held as late as February 24, although disease transmission was well underway in Europe at that time. On March 24, a national lockdown was announced with 4 hours’ notice.^5^ Although a state-level or more localized lockdown would have been preferable, the precise locations of disease hotspots were unknown because of low levels of testing.^6^ The sudden lockdown imposed a significant burden on the urban poor and migrants, who found themselves both out of work and with no means to return to their villages.

At the time of the lockdown, India had officially counted about 500 cases of COVID-19 and 10 deaths because of the contagion. The lockdown represented a law-and-order solution to a problem for which India was poorly equipped from a public health standpoint. At that time, India had approximately 1.9 million hospital beds, 95,000 intensive care unit (ICU) beds, and 48,000 ventilators, against a need of 270,000 ICU beds under an optimistic scenario of COVID-19 burden.^7^ The lockdown was essential to buy time to prepare for the eventual flood of cases.

Model-based estimates^8^ produced in March 2020 had indicated that a national lockdown could reduce the number of infections at the peak of the pandemic—expected in early May—by 70–80%, depending on the degree of public compliance with physical distancing. These projections estimated that, in the absence of any intervention, India could expect to see 5–20% of its urban population infected in a first wave.^8,9^ At an overall mortality rate of 1%, the death toll could climb to hundreds of thousands, if not higher.

By all accounts, the national lockdown was tightly enforced and has been described as one of the harshest in the world.^10^ Since late June, the lockdown has been lifted in stages and has transitioned to state-level lockdowns that have been largely reactive to local caseloads at any given time. Some form of restrictions on movement exists in most states. However, universal adherence to masking and social distancing has been difficult to enforce, and compliance has varied across states and districts. Physical distancing is not practicable in many low-income communities where there is significant crowding both within households and in public spaces. As of August, schools, colleges, movie theaters, places of worship, and most other places of mass gathering remain closed. The future outlook for reopening is unclear at this time.

**Figure 1.**
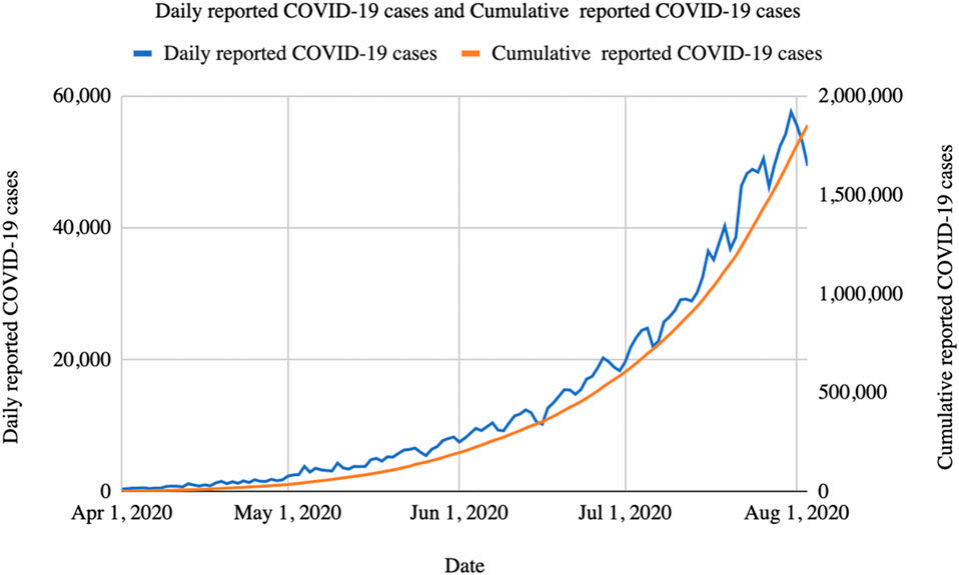
Daily and cumulative reported COVID-19 cases in India.

**Figure 2.**
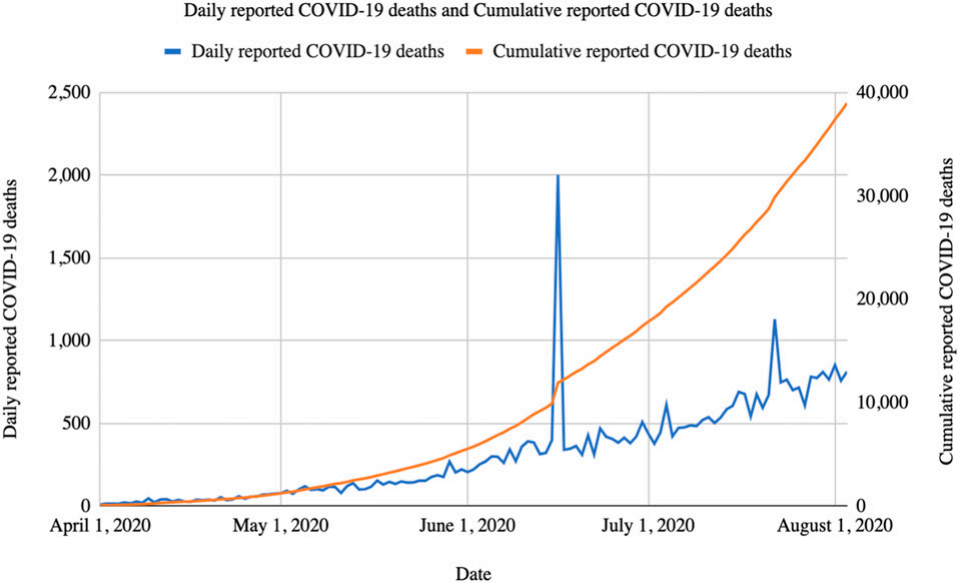
Daily and cumulative reported COVID-19 deaths in India. Note: There was a spike in the number of reported deaths on June 16, 2020 as Maharashtra alone reported around 1,400 deaths that day.

Caseloads and deaths are now increasing at the fastest rate of any large country, and India currently records more than 800 COVID-19 deaths every day (Figures 1 and 2). As of August 3, 2020, India was third in the world in the number of reported SARS-CoV-2 infections (2,456,785) and fourth in the number of reported COVID-19 deaths (48,117).^11^ Both infections and deaths are likely underestimated because of the low levels of testing in India. Mortality rates (based on reported cases and deaths) appear to be low in India, as they are in most countries in the region, perhaps indicative of both limited testing and other unexplained factors.

## MEASUREMENT OF THE EXTENT OF THE PANDEMIC

**Figure 3.**
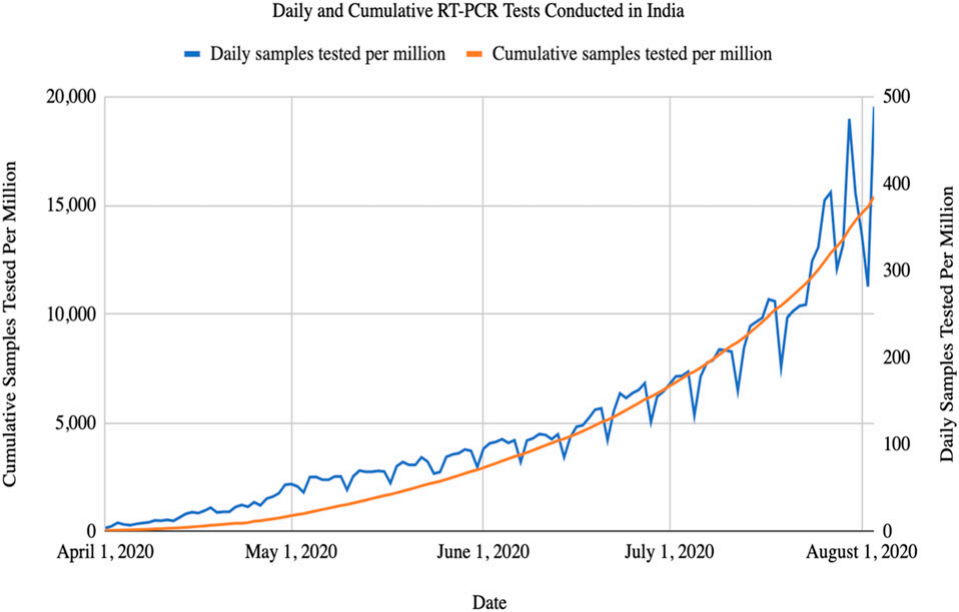
Daily and cumulative RT-PCR tests conducted in India. Data source: Media, the Indian Council for Medical Research, Ministry of Health and Family Welfare, Center for Disease Dynamics, Economics & Policy (CDDEP) calculation.

India was slow to provide testing despite significant capacity for reverse transcription-polymerase chain reaction (RT-PCR) testing in both public and private laboratories. Testing in the early days of the epidemic was limited to a few public laboratories. Private laboratories, which typically provide the bulk of pathology services, were not allowed to test at all. The restriction was not only ostensibly to maintain quality but presumably also to control information. Testing for COVID-19 in India continues to rank among the lowest in the world on a per-capita basis. Testing rose from 3,000 tests per day on March 24, 2020 (approximately 2 per million population) to more than 700,000 per day (500 per million) in August (Figure 3), although much of this increase was due to the introduction of rapid antigen tests that have far lower sensitivity than RT-PCR.^12^ At the current time, India has conducted approximately 18,000 tests per million population, a rate that is a third that of South Africa, about 60% that of Nepal, and among the lowest of any large country.

Testing rates have been highly variable across states. Daily testing in Delhi, the capital city, was around 670 per million at the end of July, comparable to that in the United States, but rates elsewhere were much lower. As of August 4, the state of Bihar was conducting just 20,000 tests per day, for a population of 105 million (190 per million). The low level of testing nationally has likely led to a large number of infections being missed. As a consequence, reported infections in India are likely indicative of only a small proportion of the total infections.

According to a serological study based on 21,387 samples conducted by India’s National CDC from June 27 to July 10, 23% of the population of Delhi had been infected.^13^ The number of seropositives—roughly 4.7 million—was approximately 40 times greater than the reported cumulative number of infections in Delhi, indicating that a large number of infections were likely missed by the testing process. Another unpublished seroprevalence study conducted in Mumbai in early July indicated that 57% of those living in slums had antibodies to SARS-CoV-2, compared with 16% of those residing in other parts of the city.^14^ Unpublished data from the Indian Council of Medical Research indicated that 10 million people (0.7% of the Indian population) had been infected through early May, at a time when fewer than 50,000 cumulative infections had been reported.^15^ Similar undercounts by a factor of 40–200 have been uncovered by comparing seroprevalence data with reported infection data. An important caveat is that the seroprevalence data have yet been published in the peer-reviewed literature.

## DISEASE TRANSMISSION AND EPIDEMIOLOGY

Despite the size of the COVID-19 problem, there has been limited published epidemiological or clinical research emerging from India. Preliminary data from comprehensive surveillance in the Indian states of Tamil Nadu and Andhra Pradesh (home to ∼128 million people) from a cohort of 4,206 index cases and 64,031 contacts found that the risk of transmission from an index case to a close contact ranged from 2.6% in the community to 9.0% in the household; these results did not differ with respect to the age of the index case.^16^ This finding indicated an important role for children and young adults in transmission, with a third of infected individuals younger than 30 years. Early analysis from this contact-tracing dataset, which represents one of the largest prospective studies of infections among exposed individuals to date, found that superspreading events were the rule, rather than the exception. Prospective follow-up testing of exposures revealed that 83% of infected individuals did not infect any of their contacts, whereas 5% accounted for 80% of observed new infections. Unlike in high-income countries, where deaths are mostly in the age-group older than 65 years, COVID-19–related deaths were concentrated at ages 50–64 years, and the incidence of reported cases did not increase with older age. Strikingly, these differences cannot be fully accounted for by differences in population age distributions. Contrary to the long hospital stays reported in high-income settings,^17^ the median time to death was 5 days following admission. The study also found substantial reductions in the reproduction number associated with the implementation of India’s countrywide lockdown.

## THE WAY FORWARD

India is a large country, and it does not face a single homogenous epidemic. Currently, 80% of cases are reported from < 10% of its districts.^18^ The epidemic is in different stages in different parts of the country, but the response has been driven by a national, overarching centralized strategy instead of being locally owned. Although opportunities for containment of infections are limited, given the tremendous economic and human cost of lockdowns, a number of measures could help reduce the mortality rate and facilitate a quicker exit from the pandemic.

1. An important aspect of COVID-19 management is averting deaths. The current national guidelines do not prioritize high-risk individuals for early testing, and this is a missed opportunity for averting deaths in vulnerable populations of the elderly and those with comorbidities.
2. Reporting of deaths is incomplete, and because many individuals die without a COVID-19 test, the number of reported deaths is likely an underestimate of the true numbers. Identification of deaths offers an opportunity to learn about the disease and, thereby, prevent future cases and deaths. A formal system of mortality surveillance, specifically to measure the additional mortality attributable to COVID-19, needs to be put in place.^19^
3. The epidemic response should be data driven and locally owned. More granular data and greater openness to data sharing and coordination would enable surveillance data to be used for management decisions, including planning regarding personal protective equipment, medicines, supplies, and, most importantly, ICU capacity and healthcare personnel. This would provide a clear picture of the impact of COVID-19 to the public and could encourage greater compliance with personal protection and distancing.
4. Nongovernmental organizations and civil society have been largely missing from the response to the pandemic and should be involved in helping mitigate the continued effects of the lockdown and enabling access to health care.
5. Guidelines for clinical protocols for patient management should be updated rapidly, consistent with global research findings, and communicated clearly to clinicians. Despite national guidelines, there is confusion about how best to care for patients at home with asymptomatic infection, in hospital with mild-to-moderate disease, with serious disease requiring high flow oxygen, and with severe disease requiring mechanical ventilation.
6. India is now in a season during which other diseases including dengue, chikungunya, malaria, and seasonal influenza have symptoms that are similar to those of COVID-19. As these diseases are likely to have overlapping spread in the country, a clinical and testing strategy to enable distinction between the diseases is needed.^20^

The COVID-19 pandemic is an opportunity to invest in the public health infrastructure of India, an area of systemic neglect over the past few decades. In the short-to-medium term, developing protocols for clinical trials to investigate candidate vaccines, drugs, and monoclonal antibodies against SARS-CoV-2 infection will be critical to ensure optimal preventive and therapeutic management of the disease, particularly to protect those at high risk of death.^21^ In the long term, a blueprint should be developed to empower and strengthen India’s national and state level mechanisms for public health research, surveillance, and policy activities. As was the case in other countries, India’s pandemic preparedness plan was largely abandoned in the face of a real pandemic. The response to COVID-19 has been driven by political priorities rather than by public health and epidemiological expertise.

Given the country’s size and its large global diaspora, India’s battle with COVID-19 will play a large role in the fate of the pandemic. As the world’s largest vaccine producer, India will likely be a major supplier of vaccines against COVID-19, if and when they are approved. The country’s largest vaccine manufacturers are gearing up to produce COVID-19 vaccines at scales that have not been attempted before. If India’s vaccine industry is successful, then it will help ensure that these vaccines will be available not only to those who can pay for them but also to the hundreds of millions of impoverished people in India and in other low- and middle-income countries who need a vaccine.

India stands at a critical juncture. Although COVID-19 is exacting a significant health and economic impact on the country, it offers an opportunity to rethink India’s approach to public health. If done correctly, the legacy of COVID-19 could be a much needed public investment in health, a well-equipped workforce to respond to future pandemics, and system capacity for surveillance, contact tracing, research, disease modeling, and response.
